# Assessment of stress and anxiety during the COVID-19 pandemic in caregivers of children with ASD

**DOI:** 10.1192/j.eurpsy.2022.712

**Published:** 2022-09-01

**Authors:** P. Pacheco, M. Pacheco, D. Molini-Avejonas

**Affiliations:** 1 University of São Paulo, Rehabilitation Science, São Paulo, Brazil; 2 UFES, Morphology, Vitoria, Brazil

**Keywords:** autism, coping, COVID19, Stress

## Abstract

**Introduction:**

An infectious disease such as COVID-19 can have a great impact on mental health due to the fear of contracting it as well as the social isolation itself due to the containment measures. Such events are considered stressors, as they can be perceived as threatening or challenging, and can have cumulative effects that are harmful to mental health. Along with this scenario, anxiety can occur in association with stress, and it is defined as extreme concern and somatic symptoms that generate tension, hindering the proper functioning and development of basic life functions. In people with Autism Spectrum Disorder (ASD) and their families, such events can occur more intensely, as changing routine and adapting to different activities are usually challenging. The study examined stress, anxiety and coping strategies during the pandemic.

**Objectives:**

To analyze stressful events, anxiety and coping strategies in caregivers of children and adolescents with ASD and typical development.

**Methods:**

Forty caregivers of children and adolescents with ASD and 40 of typically developing participated in the study. The assessment instruments used were: 1. RSQ COVID-19; 2. Semi-structured interview; 3. State–Trait Anxiety Inventory for Adults. For statistical analysis, analysis of variance (ANOVA) or chi-square were used.

**Results:**

Caregivers of children and adolescents with ASD showed greater stress and anxiety, in addition to using less adaptive coping strategies.

**Conclusions:**

There is a great need to welcome families of children and adolescents with ASD, helping to develop coping or coping strategies.

**Disclosure:**

No significant relationships.

